# Intrusive thoughts and quality of life among men with prostate cancer before and three months after surgery

**DOI:** 10.1186/1477-7525-11-154

**Published:** 2013-09-11

**Authors:** Thordis Thorsteinsdottir, Maria Hedelin, Johan Stranne, Heiddis Valdimarsdóttir, Ulrica Wilderäng, Eva Haglind, Gunnar Steineck

**Affiliations:** 1 Division of Clinical Cancer Epidemiology, Department of Oncology, Institute of Clinical Sciences, Sahlgrenska Academy at the University of Gothenburg, 41345 Gothenburg, Sweden; 2 Scandinavian Surgical Outcomes Research Group (SSORG/Gothenburg), Department of Surgery, Institute of Clinical Sciences, Sahlgrenska Academy at the University of Gothenburg, Gothenburg, Sweden; 3 Research Institute of Emergency Care, Landspitali University Hospital, Reykjavik, Iceland; 4 Division of Clinical Cancer Epidemiology, Department of Oncology–Pathology, Karolinska Institutet, Stockholm, Sweden; 5 Department of Urology, Sahlgrenska University Hospital, Gothenburg, Sweden; 6 Department of Oncological Sciences, Mount Sinai School of Medicine, New York, NY, USA; 7 Department of Psychology, Reykjavik University, Reykjavik, Iceland

**Keywords:** Prostate cancer, Quality of life, Intrusive thoughts, Clinical trial

## Abstract

**Background:**

Sudden, unwelcome and repetitive thoughts about a traumatic event – intrusive thoughts – could relate to how men assess their quality of life after prostate-cancer diagnosis. We aimed to study the prevalence of intrusive thoughts about prostate cancer and their association with quality-of-life outcomes before and after radical prostatectomy.

**Methods:**

During the first year of the LAPPRO-trial, 971 men scheduled for radical prostatectomy were prospectively included from 14 urological centers in Sweden. Of those, 833 men responded to two consecutive study-specific questionnaires before and three months after surgery (participation rate 86%). The association of intrusive thoughts with three quality-of-life outcomes, i.e. self-assessed quality of life, depressive mood and waking up with anxiety was estimated by prevalence ratios that were calculated, together with a 95% confidence interval, at the same time-point as well as over time. *Fisher’s exact*-test was used to analyze differences between respondents and non-respondents. *Wilcoxon signed-ranks* and *Cochran-Armitage trend* tests were used for analysis of change over time. To validate new questions on intrusive thoughts, written answers to open-ended questions were read and analyzed by qualitative content analysis.

**Results:**

Before surgery, 603 men (73%) reported negative intrusive thoughts about their cancer at some time in the past month and 593 men (59%) reported such thoughts three months after surgery. Comparing those reporting intrusive thoughts at least weekly or once a week before surgery with those who did not, the prevalence ratio (95% confidence interval), three months after surgery, for waking up in the middle of the night with anxiety was 3.9 (2.7 to 5.5), for depressed mood 1.8 (1.6 to 2.1) and for impaired self-assessed quality of life 1.3 (1.2 to 1.5).

**Conclusion:**

The prevalence of negative intrusive thoughts about prostate cancer at the time of surgery associates with studied quality-of-life outcomes three months later.

**Trial registration:**

Current Controlled Trials, ISRCTN06393679

## Introduction

When facing a diagnosis of a life-threatening disease such as prostate cancer, disease-related thoughts could relate to men’s assessment of quality of life. It is possible that the thoughts of men who receive a diagnosis of prostate cancer alternate between healthy reflections and involuntary thoughts. Involuntary thoughts that appear suddenly, repeatedly and that are unwelcome – intrusive thoughts – constitute one component of post-traumatic stress disorder [1] and can be seen as a marker of incomplete cognitive processing of the emotional trauma caused by the cancer diagnosis [2,3]. Intrusive thoughts have been seen as reflecting an incomplete coping process after a traumatic event [4] and, in theory, a successful coping process is vital if quality of life is to be maintained [5]. In direct relation to a traumatizing event, possibly the frequency and intensity of intrusive thoughts can serve as a measure of the quality of life. Studies performed at one time-point in the disease process or among patients receiving various treatments, suggest that there could be an association between intrusive thoughts and impaired quality of life among men with prostate cancer [4,6-9], but quality-of-life measurement scales rarely include questions on thoughts [10]. The presence of intrusive thoughts has also been linked to depression, anxiety and sleep disturbance [3,11]. On the other hand, prospective large-scale studies in well-defined study-groups could reveal if the association between intrusive thoughts and quality-of-life outcomes is sufficiently well based for further utilization in studies or clinical interventions.

Quality of life is often seen as a multi-dimensional construct and measured as such. Long-lasting physical symptoms are frequent after prostate-cancer surgery and appear to diminish the level of quality of life [12]. The development of minimally invasive surgery with the aim to minimize long-lasting physical symptoms such as urinary leakage and erectional dysfunction is ongoing [13], and thus the psychological aspects of prostate-cancer diagnosis may become more evident. By detecting factors that could be modified there could be an opportunity to improve men’s quality of life in the long run. Intrusive thoughts are a psychological symptom that seems modifiable with simple clinical interventions [14-16].

Men participating in our pre-study interviews spontaneously described their thoughts about the disease when asked about their experience after receiving the diagnosis. Based on these interviews and previous studies, we hypothesized that a higher prevalence and more intrusion of thoughts about the prostate-cancer diagnosis and treatment would be associated with higher prevalence of waking up in the middle of the night with anxiety, higher prevalence of depressed mood and with self-reports of impaired quality of life, at the same time-point and longitudinally. The aim of the study was to determine the prevalence of intrusive thoughts about prostate cancer and their association with quality-of-life outcomes before and three months after radical prostatectomy.

## Patients and methods

All men diagnosed with prostate cancer scheduled for surgery at 14 urological departments in Sweden were prospectively included to the LAPPRO-trial (ISRCTN06393679) [13,17]. The trial-secretariat monitored and collected data from consenting participants as a neutral third party [18], applying a detailed process of contacting all patients at follow-up to ascertain a high participation rate. The study design has been described in detail elsewhere [13]. The Regional Ethical Review Board in Gothenburg approved the trial (reference number 277–07).

The present study-cohort consisted of all men included in LAPPRO who underwent prostatectomy between September 1, 2008 and August 31, 2009, and who had answered two consecutive questionnaires, i.e. before and three months after surgery. Participants completed the first questionnaire at admission in the hospital. The second questionnaire was sent to the participants and completed at home. Data was analyzed regardless of surgical technique used. Prior to answering the second questionnaire participants had been informed about the pathology report on the resected prostate.

### Validation process

Before the study start, the study variables were defined, developed and validated by a mixed-methods approach. The study-specific questionnaires were based on in-depth interviews with prostate cancer patients, on measures used in research on post-traumatic stress disorder [1] as well as on previous questionnaires developed within our research group [12,19,20]. Firstly, transcriptions of the in-depth interviews were analyzed hierarchically and emerging themes were turned into questions. Then, the new questions were integrated with existing measurement-scales in order to make the study-questionnaires suitable for the study population and the time of administration. To ensure validity, specialized nurses, psychologists, clinical scientists and urologists reviewed the questionnaires for their content. After that the questionnaires were validated face-to-face with 15 prostate cancer patients: a researcher was present when each man answered the questionnaires, making sure the questions were measuring what they were meant to and that they applied to the man’s situation at that time. This resulted in further modifications, and in the last preparatory step the questionnaires were tested in a pilot-study of 100 men with consequent revisions before the main study was initiated. This validation procedure has previously been described in detail [13,21].

### Questionnaires

Intrusive thoughts were measured by the question: “How often during the past month have you had negative thoughts about your prostate cancer, suddenly and unintentionally?”. The response categories were: “Never”, “More seldom than once a week”, “At least once a week”, “At least three times a week”, “At least once a day”, “At least three times a day”, “At least seven times a day”. With the aim to validate and get a description of the participants’ thoughts, an open-ended question followed: “Freely describe with a few words your negative thoughts about prostate cancer”. Identical questions were then asked about positive thoughts about prostate cancer. Intrusion was measured by: “How intrusive have you experienced the sudden thoughts about your prostate cancer, positive as well as negative?” with the response categories: “Irrelevant, I have not had any sudden thoughts about prostate cancer”, “Not at all intrusive”, “A little bit intrusive”, “Moderately intrusive”, “Very intrusive”.

Three outcome measures indicating the men’s psychological health were chosen as these have been regarded as valid and sensitive indicators of impaired quality of life in similar study groups [12,20,22,23]. Self-assessed quality of life was measured by: “How would you describe your quality of life during the past month?” answered on a seven-point visual digital scale anchored by “0 – 6”, “0” meaning “No quality of life” and “6” “Best possible quality of life”. The prevalence of depressive mood was assessed by: “Have you been feeling depressive during the past month?” with a seven-point answering scale, “0” meaning “Never” and 6 “All the time”. Waking up with anxiety or worry was detailed by: “Have you in the past month woken up in the night with nervousness, anxiety or feeling of discomfort?” with the response categories: “No”, “Yes, but more seldom than once a week”, “Yes, at least once a week”, “Yes, at least three times a week”, “Yes, every night”.

### Statistical methods

The prevalence of thoughts about prostate cancer and their degree of intrusion were seen as the explanatory (independent) variables, while the self-assessed quality of life, depressive mood and waking up with anxiety were the explained (dependent) variables (symptoms of impaired quality of life). In the analysis, the dependent variables were dichotomized as present or not by considering the clinical significance of the symptom in question, as has been done in our previous studies [12,22,23]. Hence, the symptom “waking up with worry or anxiety” was defined to be present if the prevalence was “At least once per week”, “depressive mood” if the answers were from “Sometimes” to “All the time” and “impaired self-assessed quality of life” if answers were from “Low” to “Moderate”. Since the questions on intrusive thoughts were newly designed, the associations with the dependent variables were analyzed using two separate cut-off points, “At least once a week” and “At least three times a week”.

The percentages of the participants reporting intrusive thoughts responding within each response category (present/not present) of the dependent variables were calculated and the prevalence ratio is the quotient of those two percentages. We report prevalence ratios together with 95% confidence intervals. Differences between respondents and non-respondents, i.e. those answering only one questionnaire out of two, were analyzed with *Fisher’s exact*-test. Changes in the prevalence of thoughts between the two time-points were analyzed with *Wilcoxon matched pairs-signed* test and trends with *The Cochran-Armitage trend* test. The statistical analyses were performed in SAS 9.2 (SAS Institute Inc., Cary, NC). Patients not responding to a question were not included in the analyses of that specific variable. The written answers to each open-ended question were transcribed into Microsoft® Word and then pasted into an Excel® file. The first author read answers from 200 men with the aim to understand and validate the respondents’ use of the phenomenon intrusive thoughts as stated in the study questionnaires. The methodology of qualitative content analysis was set as ground and the text analyzed hierarchically. First, common themes in the answers were identified and then inductively coded into response categories that described different contents of the written answers [24].

## Results

During the first year of the trial, 971 participants in the LAPPRO-trial underwent radical prostatectomy. Of this group, 833 (86%) returned both questionnaires. Table 1 shows the participants’ demographics. Questionnaires from 63 participants were not collected at the urological departments before surgery because of logistic failures. Fifty-six men received but did not return either questionnaire without the secretariat knowing why; nine did not answer because of not feeling well psychosocially, another five because of not feeling well physically, while five had other reasons for not responding. The age of non-participants (mean age 62.1 years, SD 6.9, median 63, min 43, max 76, inter-quartile range 59–67 was not different from the participants (mean age 62.6 years, SD 6.2, median 63, min 41, max 77, inter-quartile range 59–67), neither was the distribution of clinical tumor stage between the groups.

**Table 1:** Characteristics of participants (n = 833) undergoing surgery for prostate cancer between September 1, 2008 and August 31, 2009

**Characteristics**	**Number**	**Percent**
Non-participants in the present cohort, out of 971 eligible	138	14.1
Not returning any questionnaire	46	4.6
Not returning questionnaire before surgery	72	7.4
Not returning questionnaire 3 months after surgery	20	2.1
Participants in the present cohort (returning two questionnaires)	833	85.9
Age quartiles (mean 62.6; SD = 6.2)		
41–59 years	230	27.6
60–77 years	603	72.4
Mean number of days before surgery, first questionnaire (median 6; SD = 27.8)	11	
Mean number of days after surgery, second questionnaire (median 91; SD = 22.8)	93	
**Level of education**		
Primary school	160	19.2
Secondary school, 3 years	238	28.6
Upper secondary school	106	12.7
College/University	317	38.1
Other	8	1.0
Not indicated	4	0.5
**Marital status**		
Married or living with partner	698	83.8
Living alone, but has partner	64	7.7
Living alone, no partner	66	7.9
Not indicated	5	0.6
Widower^a^	22	2.6
**Employment status before surgery**		
Employed	463	55.6
Unemployed	4	0.5
Retired	298	35.7
On short or long-term sick leave	38	4.6
Other	22	2.7
Not indicated	8	0.9
**Residence**		
Rural	118	14.2
Village or town	281	33.7
City (population > 500,000)	425	51.0
Abroad (not in Sweden)	5	0.6
**Use of anti-depressants**	36	4.3
**Bodily pain**, at least once a week or more	161	19.3
**Intercurrent illnesses**, one or more (yes)	417	50.1
**Clinical stage**, rectal palpation before surgery		
T1	450	54.0
T2/T3	332	39.9
Not indicated	56	16.9

^a^ 14 answers were missing on this question, 22 responded yes, 797 no. Respondents may have been widowers with a current partner.

Before surgery, 73% of the respondents reported having negative intrusive thoughts about their prostate cancer diagnosis at some time in the past month and 59% reported intrusive thoughts when asked three months after surgery (Table 2), the decline in prevalence was statistically significant (*p* < 0.0001). The prevalence before and after surgery did not differ from results from non-participants answering one questionnaire out of two.

**Table 2:** Results from 833 men asked about intrusive thoughts about prostate cancer or its treatment and symptoms of quality of life before and 3 months after surgery for prostate cancer

** Questions and response categories**	**Before surgery**	**3 months after surgery**
**How often in the past month have you had negative thoughts about your prostate cancer, suddenly and unintentionally?**	**Frequency (%)**	**Frequency (%)**
Never	226 (27)	338 (41)
Less than once a week	299 (36)	304 (37)
At least once a week	127 (15)	105 (13)
At least three times a week	71 (9)	46 (6)
At least once a day	63 (8)	30 (4)
At least three times a day	32 (4)	6 (1)
At least seven times a day	11 (1)	2 (0)
Not indicated	4 (00)	2 (0)
**How often in the past month have you had positive thoughts about your prostate cancer, suddenly and unintentionally?**		
Never	493 (59)	375 (45)
Less than once a week	130 (16)	183 (22)
At least once a week	94 (11)	126 (15)
At least three times a week	54 (7)	79 (10)
At least once a day	38 (5)	47 (6)
At least three times a day	11 (1)	9 (1)
At least seven times a day	2 (0)	6 (1)
Not indicated	11 (1)	8 (1)
**How intrusive have you experienced the sudden thoughts, positive as well as negative, about your prostate cancer?**		
Not relevant, did not have any	190 (23)	242 (29)
Not intrusive at all	210 (25)	318 (38)
A little bit intrusive	256 (31)	182 (22)
Moderately intrusive	112 (14)	70 (8)
Very intrusive	60 (7.)	15 (2)
Not indicated	5 (1)	6 (1)
**How would you describe your quality of life during the past month?**		
No quality of life = 0	1 (0)	1 (0)
1	15 (2)	8 (1)
2	37 (4)	46 (6)
3	107 (13)	133 (16)
4	215 (26)	261 (31)
5	299 (36)	269 (32)
Best possible quality of life = 6	157 (19)	113 (14)
Not indicated	2 (0)	2 (0)
**Have you, sometime during night, woken up with nervousness, anxiety or feeling of discomfort during the past month?**		
No	431 (52)	552 (66)
Yes, but more seldom than once a week	169 (20)	153 (18)
Yes, at least once a week	111 (13)	67 (8)
Yes, at least three times a week	82 (10)	41 (5)
Yes, every night	35 (4)	20 (2)
Not indicated	5 (1)	0 (0)
**Have you been feeling depressive during the past month?**		
Never = 0	135 (16)	236 (28)
1	220 (26)	225 (27)
2	156 (19)	131 (16)
3	131 (16)	103 (12)
4	119 (14)	87 (10)
5	59 (7)	45 (5)
All the time = 6	9 (1)	5 (1)
Not indicated	4 (1)	1 (0)

Before surgery, the associations of reporting negative intrusive thoughts, both with prevalence at least once a week and at least three times per week, with all the three symptoms of impaired quality of life were statistically significant on 95% confidence level as compared to those not reporting negative intrusive thoughts (Table 3). For example, the prevalence of waking up with worry or anxiety more than once per week was four times more common among men who reported negative thoughts at least once per week compared to men reporting negative thoughts less than once per week (prevalence ratio (PR) 4.0, 95% confidence interval (CI) 3.1-5.1). These associations were also statistically significant prospectively, that is reporting negative intrusive thoughts before surgery was associated with three months after surgery reporting waking up during the night (PR 3.9, 95% CI 2.7-5.5), depressive mood (PR 1.8, 95% CI 1.6-2.1) and low or moderate quality of life (PR 1.3, 95% CI 1.2-1.5). Reporting a little bit or more intrusion of thoughts was statistically significantly associated with all of the three symptoms of impaired quality of life at the same time-point both before and three months after surgery. Further, the intrusion level before surgery was statistically significantly associated with waking up with anxiety and with depressive mood three months after surgery.

**Table 3:** Prevalence ratios with corresponding 95% confidence interval (CI) for prevalence and intrusion of thoughts about prostate cancer before surgery by symptoms of quality of life before and after prostatectomy among 833 men

**Before surgery**	**Quality-of-life symptoms**^**c**^					
	**Before surgery**			**3 months after surgery**		
	**Waking up with worry or anxiety**	**Depressive mood**	**Self-assessed quality of life**	**Waking up with worry or anxiety**	**Depressive mood**	**Self-assessed quality of life**
	***≥ 1 per week***	***Sometimes to all the time***	***Low to moderate***	***≥ 1 per week***	***Sometimes to all the time***	***Low to moderate***
Negative thoughts ≥ 1 per week	157 (52%)	245 (81%)	189 (62%)	87 (29%)	188 (62%)	195 (64%)
Negative thoughts < 1 per week	69 (13%)	227 (43%)	184 (35%)	39 (7%)	180 (34%)	250 (48%)
*Prevalence ratio (95% CI)*^a^	**4.0** (3.1-5.1)	**1.9** (1.7-2.1)	**1.8** (1.5-2.0)	**3.9** (2.7-5.5)	**1.8** (1.6-2.1)	**1.3** (1.2-1.5)
Negative thoughts ≥ 3 per week	103 (59%)	146 (83%)	115 (65%)	57 (32%)	111 (63%)	109 (62%)
Negative thoughts < 3 per week	123 (19%)	326 (50%)	258 (40%)	69 (11%)	257 (39%)	336 (52%)
*Prevalence ratio (95% CI)*^a^	**3.1** (2.5-3.8)	**1.7** (1.5-1.8)	**1.6** (1.4-1.9)	**3.0** (2.2-4.1)	**1.6** (1.4-1.8)	**1.2** (1.0-1.4)
Positive thoughts ≥ 1 per week	69 (35%)	127 (64%)	79 (40%)	41 (21%)	99 (50%)	101 (51%)
Positive thoughts < 1 per week	155 (25%)	342 (55%)	292 (47%)	85 (14%)	267 (43%)	341 (55%)
*Prevalence ratio (95% CI*) ^a^	**1.4** (1.1-1.8)	**1.2** (1.0-1.3)	0.8 (0.7-1.0)	**1.5** (1.1-2.1)	1.3 (1.0-1.8)	0.9 (0.8-1.1)
A little bit intrusive or more ^b^	191 (45%)	324 (76%)	251 (59%)	99 (23%)	236 (55%)	255 (60%)
Not at all intrusive ^b^	19 (9%)	86 (41%)	64 (30%)	13 (6%)	74 (36%)	108 (51%)
*Prevalence ratio (95% CI)*^a^	**5.0** (3.2-7.7)	**1.9** (1.6-2.2)	**1.9** (1.5-2.4)	**3.7** (2.1-6.5)	**1.6** (1.3-1.9)	1.2 (1.0-1.4)

^a^ Prevalence ratios in bold style are statistically significant on 95% confidence level.

^b^ Answers only from those reporting intrusive thoughts (positive or negative) are indicated.

^c^ Waking up with discomfort or anxiety at least once a week or more frequently in the last month. Depressive mood is the highest four (2–6) on a scale of six. Low to moderate self-assessed quality of life is the lowest four (0–4) on a scale of six. All measures are within the time frame “during the past month”.

**Figure 1:**
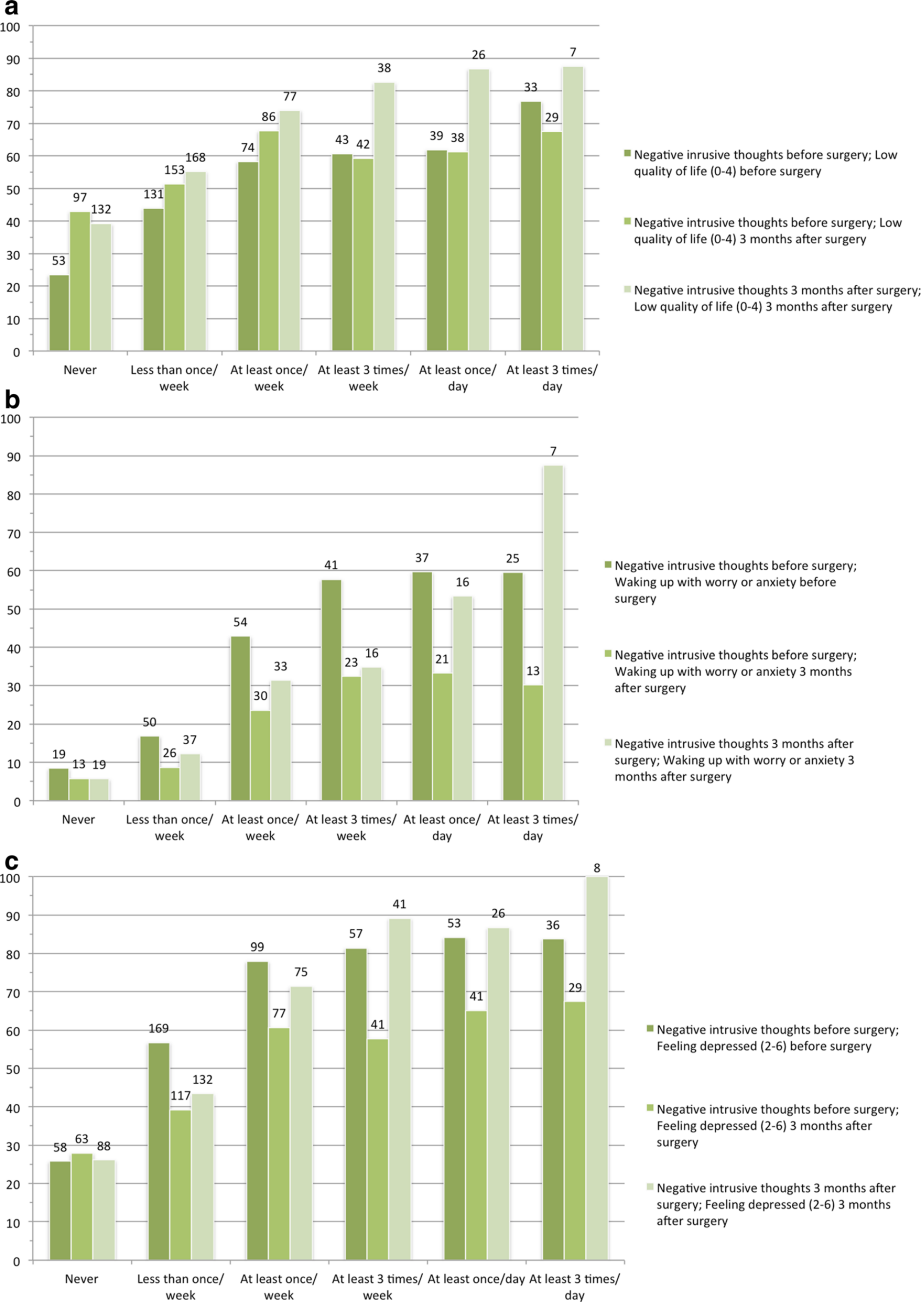
**The percentage of men within each response category of negative intrusive thoughts: (a) reporting quality of life 0–4 on the scale from 0 to 6; (b) waking up in the middle of the night with worry or anxiety at least once a week; (c) reporting the prevalence of depressive mood 2–6 on a scale from 0 to 6.** Within each category the first bar represents both measures before surgery; the second negative intrusive thoughts before surgery and the outcome after surgery, respectively; the third bar shows both measures after surgery.

The longitudinal analyses showed a statistically significant trend towards a higher percentage of men reporting symptoms of impaired quality of life as the prevalence of negative intrusive thoughts increased (*p* < 0.0001) (Figure 1).

Before surgery, 411 men (49%) described their negative thoughts about prostate cancer in free writing, as did 314 men (38%) after surgery. For positive thoughts, the respective numbers were 324 (39%) and 326 (39%). By qualitative content analysis of the text, four categories of themes (existential, emotional, preparedness, mistrust) describing negative thoughts and four categories (existential, relief, support, surgical care) for positive thoughts were identified. These were considered to describe the phenomenon intrusive thoughts as well as to validate the newly designed questions and are revealed in Table 4.

**Table 4:** Response categories of the themes repeatedly occurring in men’s expressions of positive and negative thoughts about prostate cancer before surgery based on a qualitative content analysis of written responses to open-ended questions

**Freely describe your *positive *thoughts about prostate cancer**		
**Response categories according to content analysis**	**Subject themes in responses**	**Examples of written expression**
Existential	• Positive change of life	“I can be positive to the fact that I know. The truth is easier than the lie, self-illusion. The hope for a continued meaningful life exists.”
	• Enjoy the present	“A new experience that I can learn from… about myself or others and the terms of life. Maybe I will enjoy life and others and myself even better. Live healthier – avoid stress.”
	• Overcome	
Relief	• Not spread (advanced)	“… that it was detected at an early stage and is not aggressive.”
	• Found in time	
	• Finally some results	
Support	• Family and friends	“There are many who are praying for me. Have got so much encouraging response from those around me.”
	• Other’s experience	“Four of my colleagues have had it and recovered.”
	• Good information	
Surgical care	• Symptom relief	“That I will be fine and will not need to go up and pee three times per night.”
	• Cure of cancer	“After surgery it will be cured.”
	• Regaining health	
	• Faith in surgeon	
	• Faith in surgery	
**Freely describe your *negative *thoughts about prostate cancer**		
**Response categories according to content analysis**	**Subject themes in responses**	**Examples of written expression**
Existential	• Quality of life	“Why me?”
	• Death	“Why does this happen to me? Me who is always so careful? What has made the cancer grow?”
	• The future	“The fear that it will end with me dying and leave the family with the grief.”
	• Health	
Emotional	• Fright	“Worried about insufficiencies, loneliness, fragility, impotence, to be regarded as unpleasant.”
	• Anger	
	• Worry	
	• Anxiety	
	• Uncertainty	
Preparedness	• Relative affected	“My brother had a tough ride”
	• Sneaky disease	“My father died of it at the same age as I am now.”
	• “Cancer”	“… don’t really understand why and how it has appeared.”
	• Incomprehensible	
	• Premature	
Mistrust	• Fail to be cured	“Occasionally, even a routine operation fails…”
	• Disbelief	“.. I think that the doctor is just comforting me when he is saying I will be cured.”
	• Pessimistic information	
	• Treatment choice	

## Discussion

**Figure 2:**
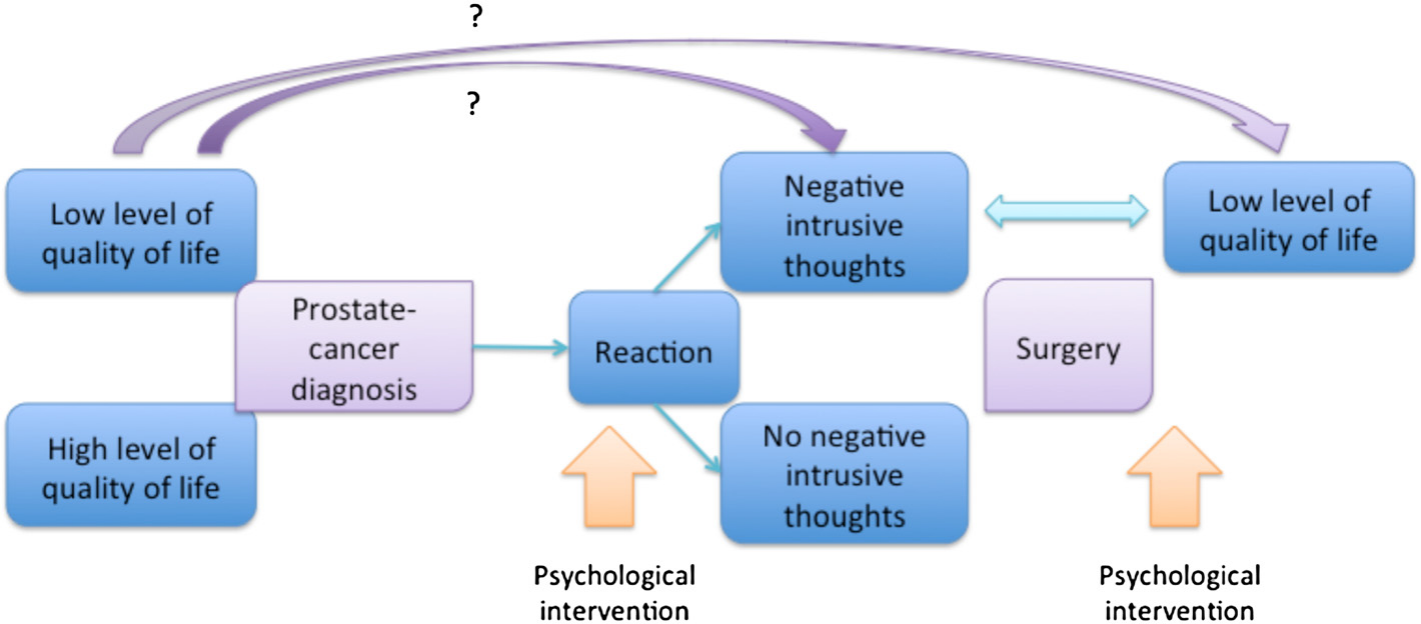
**The hypothesized association of the studied phenomena.** Suitable moments for psychological interventions are proposed. The question marks indicate possible confounding effects.

Studying men’s thoughts after prostate-cancer diagnosis may provide deeper insights into quality-of-life outcomes for the growing number of men receiving the diagnosis and having surgery than the information available from traditional quality-of-life measurement scales. In this prospective cohort study, men’s negative intrusive thoughts about prostate cancer before surgery were associated with psychological symptoms as well as with impaired self-assessed quality of life three months later. We do not know to what extent the negative intrusive thoughts led to symptoms of impaired quality of life or to what extent the impaired quality of life resulted in more negative intrusive thoughts, i.e. in which direction the associations go (Figure 2). However, this study is probably the largest addressing the question of intrusive thoughts. The association of intrusive thoughts prior to surgery with symptoms of impaired quality of life three months after surgery may inspire others to study these associations further and to design clinical interventions ultimately aiming at increasing the level of quality of life for men with prostate-cancer diagnosis.

That the men in our study who reported negative intrusive thoughts had more depressive mood and woke up during night with anxiety more often than men who did not report such thoughts is similar to the few comparable study-findings. In a cross-sectional study of 130 British men with prostate cancer at different stages and under different treatment regimens, half of the studied men reported a high level of anxiety symptoms and 18 out of those reported significantly more intrusive thoughts than non-anxious patients [25]. In addition, Roberts and colleagues, who studied 89 men 7 to 120 days after the initial treatment, mostly surgery, for localized prostate cancer, reported a correlation between lower prevalence of intrusive thoughts and better mental as well as physical functioning [7]. A similar correlation was also found in a cross-sectional study of Australian cancer outpatients (41% men) about two years after their diagnosis [26]. As our study addresses the relationships both with cross-sectional design as well as longitudinally, it confirms the impact of intrusive thoughts on quality of life that previously published studies had indicated.

A prospective association between men’s intrusive thoughts and psychological distress was found by Macefield and colleagues during prostate-specific antigen (PSA) testing and the subsequent biopsy [27]. They found that intrusive thoughts about the specific event in the testing process was associated with reporting anxiety before biopsy, but also after receiving negative biopsy results, although general distress declined after such results as compared to before biopsy. On the other hand, no association with depressive mood was found [27]. Negative intrusive thoughts could therefore be seen as a sensitive symptom relating to anxiety and depressive mood in distressing circumstances during the prostate-cancer trajectory, such as before surgery, but these symptoms do not necessarily overlap. Negative intrusive thoughts could in this manner be a clinically relevant phenomenon. The prospective relationship between reporting intrusive thoughts prior to surgery and symptoms of impaired quality of life three months later gives reason to examine if an intervention at the time of surgery might prevent the negative intrusive thoughts from becoming persistent, and if men’s quality of life could possibly be improved by such measures.

Our one-question measures can be considered as a strength for the study. The principle of “one question – one phenomenon” that our questionnaires follow strives for a clear definition of concepts. Single questions on psychological distress and quality of life have appeared to have similar sensitivity as multi-item measurement-scales [28-30]. Additionally, applying self-assessed measures was an attempt to avoid discrepancy resulting from researcher-assessed measurements [31]. Another strength of this study is the prospective design with consecutive registration of men from a homogenous population in a real-life situation during one calendar year in a, by and large, population-based setting. This, as well as the participants being generally healthy, with clinically localized cancer and restricted to surgical treatment, limits selection-induced problems. Further, we employ clinical epidemiological methods as transferred to this field by the hierarchical step-model [21] aiming at identifying and meeting possible problems in study design, data collection and interpretation of results. Attempting to minimize possible measurement errors, we first analyzed the narratives of men having experiences comparable to the proposed study group. Then, we designed questions based on their wordings and validated them in an analogous population as well as in the questionnaires with open-ended questions. The statistical associations obtained are unlikely to be explained by other confounding factors, non-participation or measurement errors. There are some limitations to the study. Firstly, the two study-questionnaires were answered by 86% of the eligible men; we do not know if the association between occurrence of intrusive thoughts and the symptoms of impaired quality of life differs among the 14% of men not participating. Secondly, the generalizability of the results to countries other than Sweden may be compromised by cultural-specific reactions to the prostate cancer diagnosis. Thirdly, the study variables were assessed with one-question measures, which may explain why our levels of intrusive thoughts differed from those reported by others. Mehnert and colleagues’ study of 511 ambulatory prostate-cancer survivors, two weeks to 141 months after their prostatectomy, revealed that 15% reported one or more intrusive symptoms (i.e. thoughts, dreams, memories) during the last month [6], which is about two thirds of the prevalence we found after surgery. A single question, despite being conceptually clear, may give a higher degree of noise than when items are summarized to form a latent variable such as for intrusive thoughts. This noise may influence prevalence figures as compared to alternative ways of asking but tend on average to dilute the effect measures for the associations between occurrence of intrusive thoughts and quality-of-life symptoms. Thus, the noise introduced by our use of single questions probably does not explain the associations we observed.

In this study, including 833 men scheduled for prostate-cancer surgery, we found that negative intrusive thoughts were associated with reporting depressive mood, waking up during night with anxiety and impaired self-assessed quality of life. This finding raises questions about other factors related to health or care possibly influencing the occurrence of negative intrusive thoughts among prostate-cancer patients and thereby having a role in the reaction after receiving such a diagnosis. Reports of severe psychological distress after the diagnosis of prostate cancer [32-35] emphasize the importance of identifying harmful and persisting reactions after receiving such news. Inspired by these results and from other cancer patients [14-16,36] health-care professionals could consider designing further studies and interventions for men with prostate cancer regarding the impact of negative intrusive thoughts on quality of life.

## Competing interests

No competing interest declared. The authors alone are responsible for the content and writing of the manuscript.

## Authors’ contributions

TT, HV, EH, and GS designed the study. TT, JS, EH, and GS collected the data. TT, MH, HV, UW, EH, and GS analyzed the data, and TT, MH, JS, HV, EH, and GS interpreted the data. UW did the statistical analysis. All authors were involved in the writing or revision of the report. All authors read and approved the final manuscript.

## Authors’ information

Eva Haglind: Principal investigator of the LAPPRO trial.

Gunnar Steineck: Deputy principal investigator of the LAPPRO trial.
